# Comparison of abdominal ultrasound, endoscopic ultrasound and magnetic resonance imaging in detection of necrotic debris in walled-off pancreatic necrosis

**DOI:** 10.1093/gastro/gou088

**Published:** 2015-01-07

**Authors:** Surinder S Rana, Vinita Chaudhary, Ravi Sharma, Vishal Sharma, Puneet Chhabra, Deepak K Bhasin

**Affiliations:** Department of Gastroenterology, Post Graduate Institute of Medical Education and Research (PGIMER), Chandigarh, India

**Keywords:** endoscopic ultrasound, abdominal ultrasound, magnetic resonance imaging, pancreatic necrosis, acute pancreatitis

## Abstract

**Background:** Walled-off pancreatic necrosis (WOPN) is an important complication of acute pancreatitis that is diagnosed by imaging modalities such as endoscopic ultrasound (EUS) or magnetic resonance imaging (MRI), which can clearly visualize necrotic debris. The effectiveness of abdominal ultrasound (USG) in detecting solid debris in WOPN is not clear.

**Methods:** A total of 52 patients (37 males, mean age 38.9 ± 12.6 years) with symptomatic WOPN were prospectively studied using EUS, MRI and USG. These investigations were done at a mean of 11.7 ± 5.5 weeks of onset of acute pancreatitis and within two days.

**Results:** WOPN was detected by EUS & MRI in all patients, whereas USG could not detect it in 4 (7.6%) patients (3 had predominantly solid WOPN, whereas one had air foci in WOPN). USG, MRI and EUS could detect solid debris in all patients with detectable WOPN. EUS and USG detected <10% debris in 10 (20%) patients, whereas MRI detected <10% debris in 14 (27%) patients. EUS and USG detected 10–40% debris in 33 patients whereas MRI detected 10–40% debris in 30 (58%) patients. More than 40% debris was better characterized on EUS and MRI with both detecting >40% debris in 8 patients (15%) compared to 5 (11%) patients having >40% debris on USG. EUS detected collaterals around WOPN that were not detected on USG or MRI.

**Conclusion:** USG can characterize the majority of WOPN, with accuracy comparable to that of EUS/MRI. However its limitations are the inability to detect collaterals around WOPN and characterize collections with high solid content or air.

## Introduction

Acute pancreatitis (AP) is a potentially life-threatening condition that is associated with local and systemic complications [1, 2]. Pancreatic fluid collections (PFCs) are an important and well recognized local complication of acute pancreatitis [1, 2]. Widespread availability of cross-sectional imaging modalities, such as computed tomography (CT) and magnetic resonance imaging (MRI), have made possible better characterization and understanding of different types of PFCs. Recently, the Acute Pancreatitis Classification Working Group proposed a revision of the Atlanta Classification for PFCs following episodes of acute pancreatitis. An important criterion used in this classification was the content of PFCs, *viz.* liquid alone or solid component admixed with a varying amount of liquid content [1]. The pancreatic pseudocyst was defined as an encapsulated collection containing essentially nil or minimal solid material, whereas walled-off pancreatic necrosis (WOPN) was defined as an encapsulated collection of solid necrotic material with varying amount of liquid content.

It is important to differentiate between an acute pseudocyst and WOPN by accurate identification and characterization of the solid necrotic debris, as this has implications in management. Patients with WOPN usually require more aggressive endoscopic drainage, in contrast to simple transmural drainage, which may be adequate for treatment of pseudocysts [3–8]. Also, in our previously published paper, we showed that the morphological features of WOPNs determine the therapeutic strategy, since collections with greater amounts of solid debris require more aggressive therapeutic interventions—such as direct endoscopic necrosectomy (DEN)—as well as more endoscopic procedures, for successful clinical outcome [9]. WOPNs contain both liquid and solid necrotic debris and it is usually difficult to distinguish them from pure liquid collections on contrast-enhanced CT (CECT) [10]. MRI and endoscopic ultrasound (EUS) have been shown to be more sensitive than CECT in detecting the solid necrotic debris [10–12]. Although EUS has been shown to be an accurate investigation for the evaluation of solid debris in WOPN, it is invasive and causes patient discomfort. Trans-abdominal ultrasound (USG) is a cheap, non-invasive and widely available investigation but has not been evaluated in patients with WOPN to establish its ability to detect solid necrotic debris. We performed a study to evaluate and compare the diagnostic performances of USG, EUS and MRI in identifying solid necrotic debris in patients with WOPN.

## Methods

This was a prospective study, in which we enrolled the study subjects from among patients that had been referred to our endoscopy unit for endoscopic drainage between April 2013 and July 2014. All the enrolled patients had earlier been diagnosed with acute necrotizing pancreatitis and were now symptomatic, with a documented pancreatic fluid collection on CECT. Exclusion criteria were pregnancy, age less than 18 years, congestive cardiac failure, compromised pulmonary status or any contraindication to MRI (presence of metal implants incompatible with MRI and claustrophobia). The study was approved by the Institutional Ethics Committee and an informed written consent was obtained from the patients prior to enrolment in the study. Following inclusion in the study protocol, all subjects underwent USG, MRI and EUS within two days. The trans-abdominal USG was performed by gastroenterologists (SSR, VS or PC) who have extensive experience in abdominal ultrasound, and the images were recorded. The pancreatic MR imaging was done at 1.5 Tesla and these images were also recorded. Similarly, EUS was performed by experienced endosonographers (SSR or DKB) using a radial echoendoscope (Pentax EG-3670 URK radial echoendoscope, Pentax Corp., Japan, or GF-UE 160 radial echoendoscope, Olympus Corp., Japan) or linear echoendoscope (Pentax EG 3870 UTK, Pentax Corp., Japan, or Olympus GF-UCT180 linear echoendoscope, Olympus Corp., Japan) at a frequency of 7.5 MHz. EUS was performed with the patient in the left lateral decubitus position under conscious sedation with intravenous midazolam (dose ranging from 2.5 mg to 5 mg).

On EUS the size, as well as the detailed morphology of the PFCs, was studied with special emphasis on the presence—as well as the amount—of solid necrotic debris. The echogenic material present in the PFCs was suggestive of solid debris. Two endosonographers (SSR and DKB) separately reviewed the EUS images to quantify the solid debris in the PFCs as <10%, 10–40% and >40%. This sub-grouping of WOPN, based on the amount of solid debris, has been previously described in a separate study by our group [9, 11]. Briefly, the quantification of the solid debris was an approximate visual judgment by the endoscopist, based on evaluation of multiple images. Two experienced endosonographers (SSR and DKB) separately reviewed the EUS images to quantify the solid debris in the PFCs and the mean of their findings was taken as the final measure of solid debris in each PFC. In the event of disagreement between the two endosonographers, the images were jointly reviewed by both and their consensus opinion was recorded.

The USG and MRI images were independently interpreted by two gastroenterologists (DKB and SSR) who were blinded to the results obtained with the other imaging modalities. The echogenic material seen in the collection on abdominal ultrasound was considered as necrotic debris whereas, on MRI, the hypo-intense areas inside the collection on T2-weighted images was interpreted as solid debris. The solid material noted within the PFCs was quantified as described above. Also an attempt was made to detect the venous collaterals around the collection on the three imaging modalities.

### Statistical analysis

The descriptive analysis was performed and the results were presented as percentages for categorical variables and mean ± standard deviation (SD) for continuous variables. The number of patients with <10%, 10–40% and >40% solid necrotic debris detected by USG, MRI and EUS were compared using the Chi-squared test. A *P*-value of <0.05 was considered as significant.

## Results

**Figure 1.**
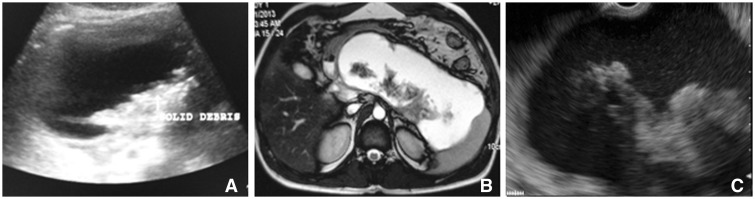
Imaging in patient with WOPN having 10–40% solid necrotic debris. a) Abdominal ultrasound; b) Magnetic resonance imaging; c) Endoscopic ultrasound.

**Figure 2.**
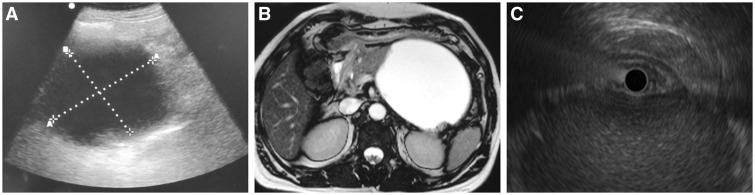
Imaging in patient with WOPN having <10 % solid necrotic debris. a) Abdominal ultrasound; b) Magnetic resonance imaging; c) Endoscopic ultrasound.

A total of fifty-two patients with WOPN were included in our study of which 37 were males with a mean age of 38.9 ± 12.6 years. All the patients had been earlier diagnosed with acute necrotizing pancreatitis and the etiology of acute pancreatitis was attributable to alcohol in 33 (63%), gall stones in 15 (29%) and idiopathic in 4 (8%) patients. The imaging (EUS, MRI and USG) was done at a mean of 11.7 ± 5.5 weeks after onset of acute pancreatitis (Figures 1 and 2). All these patients had undergone CT at the referring centre and review of the CT images revealed heterogeneous attenuation of collection suggesting solid debris in only 9 (17.3%) patients. Fourteen patients had multiple collections and the largest peri-pancreatic collection was assessed by all three imaging modalities. The mean size of WOPN was 9.3 ± 2.4 cm. The collections were detected by EUS and MRI in all patients whereas USG could not detect WOPN in four (7.6%) patients (3 patients had a predominantly solid WOPN whereas one patient had air foci in WOPN). There were no complications of EUS.

On EUS collections were well visualized and the contents could be well characterized in 51 (98%) patients. The collection in one patient could not be well assessed on EUS because of presence of air in the collection. In the remaining 51 patients, solid necrotic material appearing as echogenic material could be well visualized in all the patients. On EUS, 10 patients (20%) had <10% solid content, 33 patients (64%) had solid content varying between10–40% and 8 patients (16%) had >40% solid content. Twelve (23%) patients had venous collaterals around the WOPN because of thrombosis of the spleno-portal axis.

On MRI, collections could be well visualized in all the patients with hypo-intense areas inside the collection on T2 weighted images suggestive of solid debris. The air in the collection that was noted on CT in one patient could not be appreciated on MRI. Fourteen (27%) patients had <10% solid content, 30 (58%) patients had solid content varying between10–40% and 8 patients (15%) had >40% solid content. No collaterals around the collection could be visualized on MRI in any of the patients.

On abdominal ultrasound, the WOPN could be visualized in 48 (92%) patients. Three of these patients with undetectable WOPN on USG had a predominantly solid WOPN and one patient had air within the collection. The patients with undetectable WOPN on ultrasound were imaged within 6 weeks of the onset of symptoms. On USG, 10 patients (20%) had <10% solid content, 33 patients (69%) had solid content varying between10–40% and 5 patients (11%) had >40% solid content. No collaterals around the collection could be visualized on USG in any of the patients. The number of patients with <10%, 10–40% and >40% solid necrotic debris detected by USG, MRI and EUS were comparable (*P > 0.05*; Table 1).

**Table 1.** Solid content in walled-off pancreatic necrosis on different imaging modalities (*n* = 52)

Imaging modalities	Solid content in WOPN, *n* (%)		
	<10%	10–40%	>40%
Endoscopic ultrasound^a^	10 (19.6%)	33 (64.7%)	8 (15.7%)
Magnetic resonance imaging	14 (26.9%)	30 (57.7%)	8 (15.4%)
Abdominal ultrasound^b^	10 (20.8%)	33 (68.8%)	5 (10.4%)

^a^One case could not be well assessed on EUS because of the presence of air in the collection

^b^WOPN could not be visualized in four patients, among whom three had a high content of solid debris on EUS and one had air foci within the collection.

EUS = endoscopic ultrasound; WOPN = walled-off pancreatic necrosis

## Discussion

Accurate differentiation of acute pseudocyst from WOPN is crucial in managing patients of acute pancreatitis complicated by fluid collections. The pancreatic pseudocyst contains essentially nil or minimal solid necrotic material, whereas WOPN has a varying amount of solid necrotic material. In this prospective study of 52 patients, we compared the diagnostic capabilities of USG, EUS and MRI in identifying solid necrotic debris in patients with WOPN detected on CT.

In our study, CT images revealed heterogeneous attenuation of the collection, suggesting solid debris in only 9 (17.3%) patients and this observation is similar to the results of previous studies that have shown that CT has a poor accuracy in detecting solid debris in acute peri-pancreatic collections [10–12]. On MRI, we could detect solid debris in all 52 patients and this observation is in accord with results of the earlier studies that have shown that MR imaging depicts solid debris more frequently than CT in patients with WOPN [10–12]. Recently, EUS has been shown to be the most accurate imaging modality for characterizing peri-pancreatic collections [10], and our results support this as well. Moreover, the contents of the collections could be well characterized in all the patients except one, who had air in the collection.

Importantly, we also found that USG, a cheap and widely available imaging modality, could also detect the WOPN in 92% of the study subjects. It could not detect WOPN in patients who had a collection that was predominantly solid or contained air. Also, these patients who had a predominantly solid WOPN underwent imaging within 6 weeks of the onset of symptoms.

USG, MRI and EUS could all quantify, as well as detect, the solid debris in all the patients who had detectable WOPN. There was no significant difference in the number of patients when classified by the percentage of solid necrotic debris detected by these imaging modalities. However, in patients with <40% solid debris, MRI underestimated the amount of necrosis when compared with EUS and USG. EUS and USG detected <10% solid content in 10 patients, whereas MRI detected <10% solid content in 14 patients. EUS could also diagnose venous collaterals around the collection, which could not be identified by MRI and USG. Detection of venous collaterals around the collection is important as, during drainage of these collections, inadvertent puncture of the collaterals could lead to bleeding.

USG is not meant to replace cross-sectional imaging for the diagnosis of PFCs, but can help to better evaluate the morphology, as well as contents, of the PFCs. Patients with PFCs usually undergo EUS or MRI to detect the solid necrotic debris, since standard endoscopic drainage in the presence of solid necrotic debris produces poor results. We have reported in a separate study that patients with <10% necrotic debris needed a single session of endoscopic drainage; patients with 10–40% necrotic debris needed multiple sessions of drainage for successful outcome, while patients with >40% necrotic debris required DEN or surgical necrosectomy [9, 13].

In conclusion, USG can help in the characterization of the majority of WOPNs, with accuracy comparable to that of EUS and MRI. However, it suffers from certain limitations, including inability to detect collaterals around WOPN and inability to characterize collections with high solid content or air. EUS and MRI are comparable for characterization of WOPN but EUS is more accurate for detecting peri-WOPN collaterals.

### Disclaimers

This study was presented at Digestive Disease Week 2014, Chicago, USA

### Author contributions

Surinder S Rana performed the data analysis and wrote the manuscript. Vinita Chaudhary, Ravi K Sharma, Puneet Chhabra and Vishal Sharma contributed to the data collection and analysis. Deepak K Bhasin contributed to the writing of the manuscript and the revision and editing of the article.

*Conflict of interest statement:* none declared.
